# Ewald expansions of a class of zeta-functions

**DOI:** 10.1186/s40064-016-1732-5

**Published:** 2016-02-01

**Authors:** Kalyan Chakraborty, Shigeru Kanemitsu, Haruo Tsukada

**Affiliations:** Harish-Chandra Research Institute, Chhatnag Road, Jhunsi, Allahabad, UP 211019 India; Graduate School of Advanced Technology, Kinki University, Iizuka, Fukuoka 820-8555 Japan

**Keywords:** Zeta-function, Functional equation, Modular relation, Ewald expansion, Primary 11R42; Secondary 11R11

## Abstract

The incomplete gamma function expansion for the perturbed Epstein zeta function is known as Ewald expansion. In this paper we state a special case of the main formula in Kanemitsu and Tsukada (Contributions to the theory of zeta-functions: the modular relation supremacy. World Scientific, Singapore, 2014) whose specifications will give Ewald expansions in the *H*-function hierarchy. An Ewald expansion for us are given by $H_{1,2}^{2,0}\leftrightarrow H_{1,2}^{1,1}$ or its variants. We shall treat the case of zeta functions which satisfy functional equation with a single gamma factor which includes both the Riemann as well as the Hecke type of functional equations and unify them in Theorem 2. This result reveals the *H*-function hierarchy: the confluent hypergeometric function series entailing the Ewald expansions. Further we show that some special cases of this theorem entails various well known results, e.g., Bochner–Chandrasekharan theorem, Atkinson–Berndt theorem etc.

## Background

The incomplete gamma series has a long and rich history dating back to Riemann. We begin by stating a special case of the main formula in Kanemitsu and Tsukada (2014) and state the most general modular relation which is due the third author. This is quite technical but to make the paper self-contained we have decided to include this. Then we move into stating some results involving the familiar *G*-function, gamma functions, confluent hypergeometric functions. In the last section which is the main section so to say, we consider the modular relation in different set up and show that these specializations entail some well known Ewald expansions. The Ewald expansions we consider are only those expansions which are described under the correspondence $H_{1,2}^{2,0}\leftrightarrow H_{1,2}^{1,1}$.

## The modular relation

Let $\{\varphi _h(s)\}(1\le h \le H)$ and $\{\psi _i(s)\} (1\le i \le I)$ be given by

$$\begin{aligned} \varphi _h(s)=\sum _{k=1}^{\infty } \frac{\alpha _k^{(h)}}{\lambda _k^{(h)s}} \;\text{ and }\; \psi _i(s)=\sum _{k=1}^{\infty } \frac{\beta _k^{(i)}}{\mu _k^{(i)s}}, \end{aligned}$$

with finite abscissa of absolute convergence $\sigma _{\varphi _h}$ and $\sigma _{\psi _i}$ respectively. Here $\{\lambda _k^{(h)}\}_{k=1}^\infty$, $\{\mu _k^{(i)}\}_{k=1}^\infty$ be increasing sequences of positive terms and $\{\alpha _k^{(h)}\}_{k=1}^\infty$, $\{\beta _k^{(i)}\}_{k=1}^\infty$ be complex sequences. The (processing) gamma factor is

$$\begin{aligned} \Gamma (w\,|\,\Delta )=\frac{\prod \nolimits _{j=1}^m\Gamma \left( b_j+B_jw\right) \prod \nolimits _{j=1}^n\Gamma \left( a_j-A_jw\right) }{\prod \nolimits _{j=n+1}^p\Gamma \left( a_j+A_jw\right) \prod \nolimits _{j=m+1}^q\Gamma \left( b_j-B_jw\right) } \quad (A_j,B_j>0). \end{aligned}$$

with respect to the set of coefficients

$$\begin{aligned} \Delta =\left( \begin{array}{cc} \{(a_j,A_j)\}_{j=1}^n;& \{(1-a_j,A_j)\}_{j=n+1}^p\\ \{(b_j,B_j)\}_{j=1}^m;&\{(1-b_j,B_j)\}_{j=m+1}^q \end{array} \,\right) . \end{aligned}$$

The (Fox) *H*-function is then defined by $$(0\le n\le p$$, $$0 \le m \le q$$, $$A_j,B_j>0)$$:

$$\begin{aligned} H^{m,n}_{p,q}\left( z\Big | \Delta \right) =\frac{1}{2\pi i}\int _L\Gamma (s\,|\,\Delta ) z^{-s}{\mathrm{d}}s. \end{aligned}$$

The path *L* is subjected to the poles separation conditions similar to the one given below. Let $\chi (s)$ be a meromorphic function which satisfies:

1 $$\begin{aligned} \begin{aligned}&\chi (s)\\&={\left\{ \begin{array}{ll} \sum\nolimits _{h=1}^H\frac{\prod \nolimits _{j=1}^{M^{(h)}}\Gamma \left( d_j^{(h)}+D_j^{(h)}s\right) \prod \nolimits _{j=1}^{N^{(h)}}\Gamma \left( c_j^{(h)}-C_j^{(h)}s\right) }{\prod \nolimits _{j=N^{(h)}+1}^{P^{(h)}}\Gamma \left( c_j^{(h)}+C_j^{(h)}s\right) \prod \nolimits _{j=M^{(h)}+1}^{Q^{(h)}}\Gamma \left( d_j^{(h)}-D_j^{(h)}s\right) }\, \varphi _h(s),\\ \quad \mathop {{\mathrm{Re}}}(s)>\displaystyle \max _{1\le h \le H}\left( \sigma _{\varphi _h}\right) \\ \sum\nolimits _{i=1}^I\frac{\prod \nolimits _{j=1}^{\tilde{N}^{(i)}}\Gamma \left( e_j^{(i)}+E_j^{(i)}(r-s)\right) \prod \nolimits _{j=1}^{\tilde{M}^{(i)}}\Gamma \left( f_j^{(i)}-F_j^{(i)}(r-s)\right) }{\prod \nolimits _{j=\tilde{M}^{(i)}+1}^{\tilde{Q}^{(i)}}\Gamma \left( f_j^{(i)}+F_j^{(i)}(r-s)\right) \prod \nolimits _{j=\tilde{N}^{(i)}+1}^{\tilde{P}^{(i)}}\Gamma \left( e_j^{(i)}-E_j^{(i)}(r-s)\right) }\, \psi _i(r-s),\\ \quad \mathop {{\mathrm{Re}}}(s)<\displaystyle \min _{1\le i \le I}\left( r-\sigma _{\psi _i}\right) \end{array}\right. } \end{aligned} \end{aligned}$$

$\left( C_j^{(h)},D_j^{(h)},E_j^{(i)},F_j^{(i)}>0\right)$. For brevity we write (1) as:

2 $$\begin{aligned} \begin{aligned} \chi (s)&= {\left\{ \begin{array}{ll} \displaystyle \sum \nolimits _{h=1}^H \frac{{\mathcal {M}}^{(h)} \times {\mathcal {N}}^{h}}{{\mathcal {P}}^{(h)} \times {\mathcal {Q}}^{(h)}} \varphi _h(s) \\ \displaystyle \sum \nolimits _{i=1}^I \frac{\tilde{{\mathcal {N}}}^{(i)} \times \tilde{{\mathcal {M}}}^{i}}{\tilde{{\mathcal {Q}}}^{(i)} \times \tilde{{\mathcal {P}}}^{(i)}} \psi _i(r-s) \end{array}\right. } \end{aligned} \end{aligned}$$

where the notations are obvious. The further assumptions/choices are:

1. Only finitely many poles $s_k \, (1 \le k \le L)$ of $\chi (s)$ are not poles of
  $$\begin{aligned} \frac{{\mathcal {N}}^{h}}{{\mathcal {P}}^{(h)} \times {\mathcal {Q}}^{(h)}}\;\text{ and }\;\frac{ \tilde{{\mathcal {M}}}^{i}}{\tilde{{\mathcal {Q}}}^{(i)} \times \tilde{{\mathcal {P}}}^{(i)}}. \end{aligned}$$
2. The growth condition (for reals $u_1,u_2$ with $u_1\le u \le u_2$):
  $$\begin{aligned} \lim _{|v| \rightarrow \infty } \Gamma (u+iv-s\,|\,\Delta )\,\chi (u+iv)=0. \end{aligned}$$
  Let
  $$\begin{aligned} {\mathcal {A}} = \prod \limits _{j=1}^n\Gamma \left( a_j+A_js-A_jw\right) \;\text{ and }\;{\mathcal {B}} = \prod \limits _{j=n+1}^p\Gamma \left( a_j-A_js+A_jw\right) . \end{aligned}$$
  Also,
  $$\begin{aligned} {\mathcal {C}} = \prod \limits _{j=1}^m\Gamma \left( b_j-B_js+B_jw\right) \;\text{ and }\; {\mathcal {D}} = \prod \limits _{j=m+1}^q\Gamma \left( b_j+B_js-B_jw\right) . \end{aligned}$$
3. $L_1(s)$ is a path so that the poles of
  $$\begin{aligned} \frac{{\mathcal {A}} \times {\mathcal {N}}^{(h)}}{{\mathcal {B}} \times {\mathcal {P}}^{(h)}} \times \frac{1}{{\mathcal {D}} \times {\mathcal {Q}}^{(h)}} \end{aligned}$$
  lie on the right of $L_1(s)$, and those of
  $$\begin{aligned} \frac{{\mathcal {C}}\times {\mathcal {M}}^{(h)}}{{\mathcal {D}} \times {\mathcal {Q}}^{(h)}} \times \frac{1}{{\mathcal {B}} \times {\mathcal {P}}^{(h)}} \end{aligned}$$
  lie on the left of $L_1(s)$.
4.  $L_2(s)$ is chosen so that the poles of
  $$\begin{aligned} \frac{{\mathcal {C}} \times \prod \nolimits _{j=1}^{\tilde{M}^{(i)}}\Gamma \left( f_j^{(i)}-F_j^{(i)}r+F_j^{(i)}w\right) }{{\mathcal {D}} \times \prod \nolimits _{j=\tilde{M}^{(i)}+1}^{\tilde{Q}^{(i)}}\Gamma \left( f_j^{(i)}+F_j^{(i)}r-F_j^{(i)}w\right) } \times \frac{1}{{\mathcal {B}} \times \prod \nolimits _{j=\tilde{N}^{(i)}+1}^{\tilde{P}^{(i)}}\Gamma \left( e_j^{(i)}-E_j^{(i)}r+E_j^{(i)}w\right) } \end{aligned}$$
  lie on the left of $L_2(s)$, and those of
  $$\begin{aligned} \frac{{\mathcal {A}} \times \prod \nolimits _{j=1}^{\tilde{N}^{(i)}}\Gamma \left( e_j^{(i)}+E_j^{(i)}r-E_j^{(i)}w\right) }{{\mathcal {B}} \times \prod \nolimits _{j=\tilde{N}^{(i)}+1}^{\tilde{P}^{(i)}}\Gamma \left(e_j^{(i)}-E_j^{(i)}r+E_j^{(i)}w\right) } \times \frac{1}{{\mathcal {D}} \times \prod \nolimits _{j=\tilde{M}^{(i)}+1}^{\tilde{Q}^{(i)}}\Gamma \left( f_j^{(i)}+F_j^{(i)}r-F_j^{(i)}w\right)} \end{aligned}$$
  lie on the right of $L_2(s)$.

Under these conditions the ‘key-function’ ${\mathrm {X}}(z,s\,|\,\Delta )$ is defined as

3 $$\begin{aligned} {\mathrm {X}}(z,s\,|\,\Delta ) =\frac{1}{2\pi i}\int _{L_1(s)}\Gamma (w-s\,|\,\Delta )\chi (w)\,z^{-(w-s)}{\mathrm {d}}s. \end{aligned}$$

Then the following modular relation (Tsukada 2007) holds:

### **Theorem 1**

4 $$\begin{aligned} \begin{aligned}&{\mathrm {X}}(z,s\,|\,\Delta )\\&={\left\{ \begin{array}{ll} \displaystyle \sum \nolimits _{h=1}^H\sum \nolimits _{k=1}^{\infty }\frac{\alpha _k^{(h)}}{\lambda _k^{(h)s}} H_{p+P^{(h)},q+Q^{(h)}}^{m+M^{(h)},n+N^{(h)}}\left( z \lambda _k^{(h)} \, \left| \begin{array}{l} \{(1-a_j,A_j)\}_{j=1}^n,\{(1-c_j^{(h)}+C_j^{(h)}s,C_j^{(h)})\}_{j=1}^{N^{(h)}}, \\ \{(b_j,B_j)\}_{j=1}^m,\{(d_j^{(h)}+D_j^{(h)}s,D_j^{(h)})\}_{j=1}^{M^{(h)}}, \end{array}\right. \right. \\ \left. \begin{array}{l} \{(a_j,A_j)\}_{j=n+1}^p,\{(c_j^{(h)}+C_j^{(h)}s,C_j^{(h)})\}_{j=N^{(h)}+1}^{P^{(h)}} \\ \{(1-b_j,B_j)\}_{j=m+1}^q,\{(1-d_j^{(h)}+D_j^{(h)}s,D_j^{(h)})\}_{j=M^{(h)}+1}^{Q^{(h)}} \end{array} \right) \\ \text {if }L_1(s)\text {can be taken to the right of}\, \displaystyle \max _{1\le h \le H} \left( \sigma _{\varphi _h}\right) \\ \displaystyle \sum \nolimits _{i=1}^I \sum\nolimits _{k=1}^{\infty }\frac{\beta _k^{(i)}}{\mu _k^{(i)r-s}} H_{q+\tilde{Q}^{(i)},p+\tilde{P}^{(i)}}^{n+\tilde{N}^{(i)},m+\tilde{M}^{(i)}} \left( \frac{\mu _k^{(i)}}{z} \, \left| \begin{array}{l} \{(1-b_j,B_j)\}_{j=1}^m,\{(1-f_j^{(i)}+F_j^{(i)}(r-s),F_j^{(i)})\}_{j=1}^{\tilde{M}^{(i)}},\\ \{(a_j,A_j)\}_{j=1}^n,\{(e_j^{(i)}+E_j^{(i)}(r-s),E_j^{(i)})\}_{j=1}^{\tilde{N}^{(i)}}, \end{array} \right. \right. \\ \left. \begin{array}{l} \{(b_j,B_j)\}_{j=m+1}^q,\{(f_j^{(i)}+F_j^{(i)}(r-s),F_j^{(i)})\}_{ j=\tilde{M}^{(i)}+1}^{\tilde{Q}^{(i)}} \\ \{(1-a_j,A_j)\}_{j=n+1}^p,\{(1-e_j^{(i)}+E_j^{(i)}(r-s),E_j^{(i)})\}_{ j=\tilde{N}^{(i)}+1}^{\tilde{P}^{(i)}} \end{array} \right) \\ +\sum\nolimits _{k=1}^L \mathop {{\mathrm{Re}}}s\Big (\Gamma (w-s\,|\,\Delta )\chi (w)\,z^{s-w},w=s_k \Big ) \\ \text {if }L_2(s)\text { can be taken to the left of}\, \displaystyle \min _{1\le i \le I} \left( r-\sigma _{\psi _i}\right) , \end{array}\right. } \end{aligned} \end{aligned}$$

*is equivalent to the functional equation* (1) *or* (2).

## Formulas and some interesting *G*-function relations

In this section we recall some results involving the *G*-functions, gamma functions, confluent hypergeometric functions etc. We also derive/re-establish some interesting relations involving these functions.

- The confluent hypergeometric functions of the first kind is
  $$\begin{aligned} \Phi (a,c;z)={}_1F_1\left( \begin{array}{l} a\\ c \end{array} ;z \right) . \end{aligned}$$
- The $$H \rightarrow G$$ formula:
  5 $$\begin{aligned} & H^{m,n}_{p,q}\left( z\left| \begin{array}{l} (a_1,\frac{1}{C}),\ldots ,(a_n,\frac{1}{C}),(a_{n+1},\frac{1}{C}),\ldots ,(a_p,\frac{1}{C}) \\ (b_1,\frac{1}{C}),\ldots ,(b_m,\frac{1}{C}),(b_{m+1},\frac{1}{C}),\ldots ,(b_q,\frac{1}{C}) \end{array}\right. \right) \\ & \quad = C\,G^{m,n}_{p,q}\left( z^C\left| \begin{array}{l} a_1,\ldots ,a_n,a_{n+1},\ldots ,a_p\\ b_1,\ldots ,b_m,b_{m+1},\ldots ,b_q \end{array}\right. \right) \quad (C>0) \end{aligned}$$
  The case when $C =1$ is the Meijer *G*-function.
- The reduction-augmentation formula (Kanemitsu and Tsukada 2014, Chap. 2):
  6 $$\begin{aligned} \begin{aligned}&H\left( z\left| \,\Delta \oplus \left( \begin{array}{cc} (c,C);& -\\ -; & (c,C) \end{array} \right) \right.\right) \\ & \quad =H\left( z\left| \,\Delta \oplus \left( \begin{array}{cc} -;& (c,C)\\ (c,C) ;& - \end{array} \right) \right. \right) \\ & \quad =H\big (z\big |\,\Delta \big ). \end{aligned}\end{aligned}$$
- Reciprocity formula: The reciprocity formula for the gamma function and Euler’s identity lead to
  7 $$\begin{aligned} \begin{aligned}&H\left( z\left| \,\Delta \oplus \left( \begin{array}{cc} -; & (c,C)\\ -;& (c,C) \end{array} \right) \right. \right) \\&\quad =\frac{1}{2\pi i}\Big \{e^{c\pi i}\,H\big (e^{-C\pi i}z\big |\,\Delta \big ) -e^{-c\pi i}\,H\big (e^{C\pi i}z\big |\,\Delta \big )\Big \} \end{aligned} \end{aligned}$$
  Let us write (7) as:
  8 $$\begin{aligned} \begin{aligned}&H^{m,n}_{p+1,q+1}\left( z\left| \begin{array}{l} \{(a_j,A_j)\}_{j=1}^n,\{(a_j,A_j)\}_{j=n+1}^p,(c,C)\\ \{(b_j,B_j)\}_{j=1}^m,\{(b_j,B_j)\}_{j=m+1}^q,(c,C) \end{array} \right. \right) \\&\quad =\frac{1}{2\pi i}\left\{ e^{c\pi i}\,H^{m,n}_{p,q}\left( e^{-C\pi i}z\left| \begin{array}{l} \{(a_j,A_j)\}_{j=1}^n,\{(a_j,A_j)\}_{j=n+1}^p\\ \{(b_j,B_j)\}_{j=1}^m,\{(b_j,B_j)\}_{j=m+1}^q \end{array}\right. \right) \right. \\&\quad \quad -\left. e^{-c\pi i}\,H^{m,n}_{p,q}\left( e^{C\pi i}z\left| \begin{array}{l} \{(a_j,A_j)\}_{j=1}^n,\{(a_j,A_j)\}_{j=n+1}^p\\ \{(b_j,B_j)\}_{j=1}^m,\{(b_j,B_j)\}_{j=m+1}^q \end{array}\right. \right) \right\} \end{aligned} \end{aligned}$$

Then it entails *G*-function formula:

9 $$\begin{aligned} \begin{aligned}&G^{m,n}_{p+1,q+1}\left( z\left| \begin{array}{l} a_1,\ldots ,a_n,a_{n+1},\ldots ,a_p,c\\ b_1,\ldots ,b_m,b_{m+1},\ldots ,b_q,c \end{array} \right. \right) \\&\quad =\frac{1}{2\pi i}\left\{ e^{c\pi i}\,G^{m,n}_{p,q}\left( e^{-\pi i}z\left| \begin{array}{l} a_1,\ldots ,a_n,a_{n+1},\ldots ,a_p\\ b_1,\ldots ,b_m,b_{m+1},\ldots ,b_q \end{array}\right. \right) \right. \\&\quad \quad -\left. e^{-c\pi i}\,G^{m,n}_{p,q}\left( e^{\pi i}z\left| \begin{array}{l} a_1,\ldots ,a_n,a_{n+1},\ldots ,a_p\\ b_1,\ldots ,b_m,b_{m+1},\ldots ,b_q \end{array}\right. \right) \right\} \end{aligned} \end{aligned}$$

The beta transform (or the beta integral) is a special case of

10 $$\begin{aligned}&\Gamma (a-s)\Gamma (b+s): \nonumber \\&G_{1,1}^{1,1}\left( z \left| \begin{array}{c} a \\ b \end{array} \right. \right) =\Gamma (1-a+b)\,z^b\,(z+1)^{a-b-1} \end{aligned}$$

Using (10) we can relate the *G*-functions and the confluent hypergeometric series of the first kind [also compare (Erdélyi et al. 1953, (4), p. 256) or (Prudnikov et al. 1986, 8.4.45.1, p. 715)]:

11 $$\begin{aligned} G_{1,2}^{1,1}\left( z \left| \begin{array}{c}1-a \\ 0, 1-b \end{array}\right. \right) =\frac{\Gamma (b)}{\Gamma (a)}\,{}_1F_1\left( \begin{array}{@{}c@{}}a \\ b\end{array}\,;-z\right) =\frac{\Gamma (b)}{\Gamma (a)}\,\Phi (a,b;-z) \end{aligned}$$

In view of (17) the relation (11) reduces to

12 $$\begin{aligned}&G_{1,2}^{1,1}\left( z \left| \begin{array}{c} 1 \\ a,0 \end{array} \right. \right) =\Gamma (a)-\Gamma \big (a,z\big ) \end{aligned}$$

and

13 $$\begin{aligned} G_{1,2}^{2,0}\left( z \left| \begin{array}{c} a \\ b,c \end{array} \right. \right) =e^{-z}\,z^b\,U\big (a-c,b-c+1,z\big ) \end{aligned}$$

where *U*(*a*, *c*; *z*) is the confluent hypergeometric function of the second kind. One also has,

14 $$\begin{aligned} \Gamma (a,z)=e^{-z}U(1-a,1-a;z) \end{aligned}$$

(Erdélyi et al. 1953, (21), p. 266). If we take $a=1, c=0$ and change *a* for *b* then (13) reduces to

15 $$\begin{aligned} G_{1,2}^{2,0}\left( z \left| \begin{array}{c} 1 \\ a,0 \end{array} \right. \right) =\Gamma \big (a,z\big ). \end{aligned}$$

Also,

16 $$\begin{aligned}&G_{1,2}^{2,1}\left( z \left| \begin{array}{c} a \\ b,c \end{array} \right. \right) \\ & \quad =\Gamma (1-a+b)\,\Gamma (1-a+c)\,z^b\,U\big (b-a+1,b-c+1,z\big ). \end{aligned}$$

Thus the relation (21) in (Erdélyi et al. 1953, p. 266) becomes,

17 $$\begin{aligned} \Gamma (a)-\Gamma (a,z)=a^{-1}z^a\Phi (a,a+1;z). \end{aligned}$$

Now once again using (14) with $b=a$ relation (16) gives,

18 $$\begin{aligned}&G_{1,2}^{2,1}\left( z \left| \begin{array}{c} a \\ a,b \end{array}\right. \right) = \Gamma (1-a+b)\,\Gamma \big (a-b,z\big )\,e^{z}z^{b}. \end{aligned}$$

## Ewald expansion for zeta-functions with a single gamma factor

We consider the general modular relation associated to Dirichlet series that satisfy the functional equation with a single gamma factor. This includes both the type of zeta-functions which satisfy the functional equation of the Riemann and that of Hecke type.

Let us consider two Dirichlet series

$$\begin{aligned} \varphi (s)=\sum _{k=1}^\infty \frac{\alpha _k}{\lambda _k^s}, \quad \psi (s)=\sum _{k=1}^\infty \frac{\beta _k}{\mu _k^s} \end{aligned}$$

with finite abscissas of absolute convergence $\sigma _\varphi$ and $\sigma _\psi$, respectively. We assume that they satisfy the functional equation with a single gamma factor:

19 $$\begin{aligned} \chi (s)={\left\{ \begin{array}{ll} \Gamma (Cs)\varphi (s), & \quad {\mathrm{Re}}(s)>\sigma _\varphi \\ \Gamma (C(r-s))\psi (r-s),&\quad {\mathrm{Re}}(s)<r-\sigma _\psi \end{array}\right. } \end{aligned}$$

or in other way,

$$\begin{aligned}\chi(s)=\left\{ \begin{array}{ll}\Gamma\left(s\left|\begin{array}{ll} -; &-\\ (0,C ); & - \end{array} \right. \right)\varphi (s), & \quad {\mathrm{Re}} (s)>\sigma _\varphi \\ \Gamma \left(r-s\left| \begin{array}{ll} -; & - \\ ( 0,C) ; &- \end{array}\right. \right) \psi (1-s), & \quad {\mathrm{Re}} (s)<r-\sigma _\psi\end{array}\right.\end{aligned}$$

**Special cases of** (19):

1. A special case of (19) is the Dirichlet series satisfying the Riemann type functional equation with $C=\frac{1}{2}$, i.e.
  20 $$\begin{aligned} \Gamma \left( \frac{1}{2}s\right) \varphi (s)=\Gamma \left( \frac{1}{2}(r-s)\right) \psi (r-s) \end{aligned}$$
  with a simple pole at $s=r>0$with residue $\rho$. The typical example is of course the Riemann zeta-function $\zeta (s)=\zeta (s,1)$, where,
  $$\begin{aligned} \zeta (s,w)=\sum _{k=0}^\infty \frac{1}{\big (k+w\big )^{s}}, \quad {\mathop {{\mathrm{Re}}}}(s)>1 \end{aligned}$$
  is the Hurwitz zeta-function. We refer the reader to Example 15 for its functional equation.
2. Another special case is the Dirichlet series satisfying the Hecke type of functional equation with $C=1$, i.e.
  21 $$\begin{aligned} \Gamma \left( s\right) \varphi (s)=\Gamma \left( r-s\right) \psi (r-s) \end{aligned}$$
  with a simple pole at $s=r$ with residue $\rho$. Typical examples are the Dedekind zeta-function of an imaginary quadratic field or the zeta-function associated to modular forms.

Then by Theorem 1 one has the following modular relation:

$$\begin{aligned} &\sum _{k=1}^\infty \frac{\alpha _k}{\lambda _k^s}\,H\left( z\lambda _k \left| \left( \begin{array}{cc} -;&-\\ ( Cs,C);& -\end{array} \right) \oplus \Delta \right. \right) \\&=\sum _{k=1}^\infty \frac{\beta _k}{\mu _k^{1-s}}\,H\left( \frac{\mu _k}{z}\left| \left( \begin{array}{cc} -;&-\\ ( C(r-s),C); & -\end{array} \right) \oplus \Delta ^* \right. \right) \\ & \quad +\sum _{k=1}^L {\mathrm{Res}} (\Gamma (w-s\,|\,\Delta )\,\chi (w)\,z^{s-w},w=s_k ). \end{aligned}$$

Traditionally:

22 $$\begin{aligned}&\sum _{k=1}^{\infty } \frac{\alpha _k}{\lambda _k^s}\, H_{p,q+1}^{m+1,n}\left( z\lambda _k\left| \begin{array}{c} \{(1-a_j,A_j)\}_{j=1}^{n},\{(a_j,A_j)\}_{j=n+1}^{p} \\ \left( Cs,C\right) ,\{(b_j,B_j)\}_{j=1}^{m},\{(1-b_j,B_j)\}_{j=m+1}^{q} \end{array} \right. \right) \nonumber \\&=\sum _{k=1}^{\infty } \frac{\beta _k}{\mu _k^{1-s}}\, H_{q,p+1}^{n+1,m}\left( \frac{\mu _k}{z}\left| \begin{array}{c} \{(1-b_j,B_j)\}_{j=1}^{m},\{(b_j,B_j)\}_{j=m+1}^{q} \\ \left( C(r-s),C\right) ,\{(a_j,A_j)\}_{j=1}^{n},\{(1-a_j,A_j)\}_{j=n+1}^{p} \end{array} \right. \right) \nonumber \\&\quad + \sum _{k=1}^L {\mathop {{\mathrm{Res}}}}\Big (\Gamma (w-s\,|\,\Delta )\,\chi (w)\,z^{s-w},w=s_k \Big ) \end{aligned}$$

In this form and with $r=1$, the modular relation is given by the following simultaneous exchange of parameters:

$$\begin{aligned} \boxed { \begin{array}{rcl} s &{}\leftrightarrow &{} 1-s\\ z &{}\leftrightarrow &{} \frac{1}{\,z\,}\\ \big \{(a_j,A_j)\big \}_{j=1}^n &{}\leftrightarrow &{} \big \{(b_j,B_j)\big \}_{j=1}^m\\ \big \{(a_j,A_j)\big \}_{j=n+1}^p &{}\leftrightarrow &{} \big \{(b_j,B_j)\big \}_{j=m+1}^q\\ \big ( \Delta &{}\leftrightarrow &{} \Delta ^{*} \big ) \end{array} } \end{aligned}$$

The following theorem which is a special case of (22) will be helpful in deducing Ewald expansions.

### **Theorem 2**

(Tsukada 2007) *The zeta-functions which satisfy the functional equation* (19), *have a general Ewald expansion*$H_{1,2}^{2,0}\leftrightarrow H_{1,2}^{1,1}$*which is equivalent to* (19):

23 $$\begin{aligned} \begin{aligned}&\sum _{k=1}^{\infty } \frac{\alpha _k}{\lambda _k^s} \,H_{1,2}^{2,0}\left( z\lambda _k\left| \begin{array}{c} (a,A) \\ \left( Cs,C\right) ,(b,B) \end{array} \right. \right) \\&=\sum _{k=1}^{\infty } \frac{\beta _k}{\mu _k^{r-s}} \,H_{1,2}^{1,1}\left( \frac{\mu _k}{z}\left| \begin{array}{c} (1-b,B) \\ \left( C(r-s),C\right) ,(1-a,A) \end{array} \right. \right) \\&\quad +\sum _{k=1}^L {\mathop {{\mathrm{Res}}}}\left( \frac{\Gamma (b-Bs+Bw)}{\Gamma (a-As+Aw)}\,\chi (w)\,z^{s-w},w=s_k\right) \end{aligned} \end{aligned}$$

*and its special case is:*

24 $$\begin{aligned} \begin{aligned}&\frac{1}{C}\sum _{k=1}^{\infty } \frac{\alpha _k}{\lambda _k^s} \,G_{1,2}^{2,0}\left( {(z\lambda _k)}^{\frac{1}{C}}\left| \begin{array}{c} a \\ Cs,b \end{array} \right. \right) \\&=\frac{1}{C}\sum _{k=1}^{\infty } \frac{\beta _k}{\mu _k^{r-s}} \,G_{1,2}^{1,1}\left( {\left( \frac{\mu _k}{z}\right) }^{\frac{1}{C}}\left| \begin{array}{c} 1-b \\ C(r-s),1-a \end{array} \right. \right) \\&\quad +\sum _{k=1}^L {\mathop {{\mathrm{Res}}}}\left( \frac{\Gamma (b-Cs+Cw)}{\Gamma (a-Cs+Cw)}\,\chi (w)\,z^{s-w},w=s_k\right) \end{aligned} \end{aligned}$$

with $$A=B=C$$ [cf. (5)].

### Confluent hypergeometric series

In this subsection we will deal with confluent hypergeometric series and in the next result we show it imply incomplete gamma series, i.e. Ewald expansions. The notations are all as before.

#### **Theorem 3**

*The confluent hypergeometric expansion*

25 $$\begin{aligned} \begin{aligned}&\frac{1}{C}\,z^s\sum _{k=1}^{\infty } \alpha _k\,e^{-{(z\lambda _k)}^{1/C}}\,U\Big (a-b,Cs-b+1,{(z\lambda _k)}^{1/C}\Big )\\&=\frac{1}{C}{\left( z^{-1}\right) }^{r-s}\,\frac{\Gamma \left( b+C(r-s)\right) }{\Gamma \left( a+C(r-s)\right) } \sum _{k=1}^{\infty }\beta _k\,{}_1F_1\left( \begin{array}{c}b+C(r-s)\\ a+C(r-s)\end{array}\,;-{\left( \frac{\mu _k}{z}\right) }^{1/C}\right) \\&\quad +\sum _{k=1}^L {\mathop {{\mathrm{Res}}}}\left( \frac{\Gamma \left( b-Cs+Cw\right) }{\Gamma \left( a-Cs+Cw\right) }\, \chi (w)\,z^{s-w},w=s_k\right) \end{aligned}\end{aligned}$$

*gives the incomplete gamma series*

26 $$\begin{aligned} \begin{aligned}&\sum _{k=1}^{\infty } \frac{\alpha _k}{\lambda _k^s} \,\Gamma \left( Cs,{(z\lambda _k)}^{1/C}\right) \\&= \sum _{k=1}^{\infty } \frac{\beta _k}{\mu _k^{r-s}}\left( \Gamma \left( C(r-s)\right) - \Gamma \left( C(r-s),{\left( \frac{\mu _k}{z}\right) }^{1/C}\right) \right) \\&\quad +\sum _{k=1}^L {\mathop {{\mathrm{Res}}}}\left( \frac{1}{-s+w}\,\chi (w)\,z^{s-w},w=s_k \right) . \end{aligned} \end{aligned}$$

#### *Proof*

The first assertion (25) follows from (24) in view of (13) and (11). The second assertion, i.e., (26) is a special case of (25) with $a=1,b=0$ in view of (15) and (12). $\square$

The Riemann and the Hecke type of functional equations would take the following shape in the light of the previous theorem.

#### **Corollary 4**

(i) *The Riemann type functional equation* (19) *with*$C=\frac{1}{2}$*entails*
  27 $$\begin{aligned} \begin{aligned} \sum _{k=1}^{\infty } \frac{\alpha _k}{\lambda _k^s} \,\Gamma \left( \frac{s}{2},z^2\lambda _k^2\right)&= \sum _{k=1}^{\infty } \frac{\beta _k}{\mu _k^{r-s}}\left( \Gamma \left( \frac{r-s}{2}\right) - \Gamma \left( \frac{r-s}{2},\frac{\mu _k^2}{z^2}\right) \right) \\&\quad +\sum _{k=1}^L {\mathop {{\mathrm{Res}}}}\left( \frac{1}{-s+w}\,\chi (w)\,z^{s-w},w=s_k \right) . \end{aligned}\end{aligned}$$
(ii) *The Hecke type functional equation* (19) *entails*
  28 $$\begin{aligned} \begin{aligned} \sum _{k=1}^{\infty } \frac{\alpha _k}{\lambda _k^s} \,\Gamma \left( s,z\lambda _k\right)&= \sum _{k=1}^{\infty } \frac{\beta _k}{\mu _k^{r-s}}\left( \Gamma (r-s)- \Gamma \left( r-s,\frac{\mu _k}{z}\right) \right) \\&\quad +\sum _{k=1}^L {\mathop {{\mathrm{Res}}}}\left( \frac{1}{-s+w}\,\chi (w)\,z^{s-w},w=s_k \right) . \end{aligned} \end{aligned}$$

Formula (28) is stated as [Kanemitsu et al. 2002, Theorem 1, (1.6)], which is a basis for the Riemann–Siegel integral formula developed in (Kanemitsu et al. 2002, §4).

### Bochner–Chandrasekharan formula as $H_{1,2}^{2,0}\leftrightarrow H_{1,2}^{1,1}$

There are variety of specifications of (22) one of which is (24). We state one more specification which leads to the formula of Bochner and Chandrasekharan, which is of independent interest.

#### **Theorem 5**

29 $$\begin{aligned} \begin{aligned}&\frac{1}{B}\sum _{k=1}^{\infty } \frac{\alpha _k}{\lambda _k^s} {(z\lambda _k)}^{b/B}e^{-{(z\lambda _k)}^{1/B}} \\&=\frac{1}{C}{\left( z^{-1}\right) }^{r-s}\sum _{k=1}^{\infty } \beta _k \, {}_1\Psi _1 \left( \begin{array}{c} (b+B(r-s),\frac{B}{C}) \\ (Cr,1) \end{array};-{\left( \frac{\mu _k}{z}\right) }^{1/C}\right) \\&\quad +\sum _{k=1}^L {\mathop {{\mathrm{Res}}}}\left( \frac{\Gamma (b-Bs+Bw)}{\Gamma (Cw)}\,\chi (w)\,z^{s-w},w=s_k\right) . \end{aligned}\end{aligned}$$

#### *Proof*

We substitute $a=Cs$ and $A=C$ in (22) and then apply the reduction-augmentation formula (6). That would give the proof. We obtain as an intermediate the following:

30 $$\begin{aligned} \begin{aligned}&\sum _{k=1}^{\infty } \frac{\alpha _k}{\lambda _k^s} \,H_{0,1}^{1,0}\left( z\lambda _k\left| \begin{array}{c} - \\ (b,B) \end{array} \right. \right) \\&=\sum _{k=1}^{\infty } \frac{\beta _k}{\mu _k^{r-s}} \,H_{1,2}^{1,1}\left( \frac{\mu _k}{z}\left| \begin{array}{c} (1-b,B) \\ \left( C(r-s),C\right) ,(1-Cs,C) \end{array} \right. \right) \\&\quad +\sum _{k=1}^L {\mathop {{\mathrm{Res}}}}\left( \frac{\Gamma (b-Bs+Bw)}{\Gamma (Cw)}\,\chi (w)\,z^{s-w},w=s_k\right) \end{aligned} \end{aligned}$$

$$\square$$

Berndt (1969, Theorem 10.1) generalized the classical result of Szegö (1926) pertaining to Laguerre polynomials.

We derive it as Corollary [7, (iii)] of Theorem 5.

#### **Definition 6**

The *Laguerre polynomial* is defined by

$$\begin{aligned} L_{n}^{\alpha }(x)=\frac{1}{n!}e^xx^{-\alpha }\frac{{\mathrm{d}}^n}{\mathrm{d}^nx}(e^xx^{n+\alpha }) \end{aligned}$$

for $$0\le n\in {\mathbb {Z}}$$.

#### **Corollary 7**

(i) *With*$C=\frac{1}{2}$*the Riemann type equation* (19) *gives*
  31 $$\begin{aligned} \begin{aligned} 2\sum _{k=1}^{\infty } \frac{\alpha _k}{\lambda _k^s}\,e^{-z^2 \lambda _k^2}&=2\,\frac{\Gamma \left( \frac{r-s}{2}\right) }{\Gamma \left( \frac{r}{2} \right) }\,z^{s-r}\sum _{k=1}^{\infty } \beta _k \, {}_1F_1\left( \begin{array}{c} \frac{r-s}{2} \\ \frac{r}{2} \end{array}\,; -\frac{\mu _k^2}{z^2}\right) \\&\quad +\sum _{k=1}^L {\mathop {{\mathrm{Res}}}}\left( \frac{\Gamma \left( -\frac{s}{2}+\frac{w}{2}\right) }{\Gamma \left( \frac{w}{2}\right) } \,\chi (w)\,z^{s-w},w=s_k \right) \end{aligned}\end{aligned}$$
(ii) *With*$C=1$*the Hecke type functional equation* (19) *is*
  32 $$\begin{aligned} \begin{aligned} \sum _{k=1}^{\infty } \frac{\alpha _k}{\lambda _k^s}\,e^{-z \lambda _k}&=\frac{\Gamma (r-s)}{\Gamma (r)}\,z^{s-r}\sum _{k=1}^{\infty } \beta _k \, {}_1F_1\left( \begin{array}{c} r-s \\ r \end{array}; -\frac{\mu _k}{z}\right) \\&\quad +\sum _{k=1}^L \mathop {{\mathrm{Res}}}\left( \frac{\Gamma (-s+w)}{\Gamma (w)}\,\chi (w)\,z^{s-w},w=s_k \right) \end{aligned}\end{aligned}$$
(iii) *Again* (19) *with*$C=1$*and*$2\pi \lambda _k$*in place of*$\lambda _k$*entails*
  33 $$\begin{aligned}&\sum _{k=1}^{\infty } \alpha _k{(2\pi \lambda _k)}^n\,e^{-2\pi z \lambda _k} =n!\,z^{-r-n}\sum _{k=1}^{\infty } \beta _k \, L_{n}^{(r-1)}\left( \frac{2\pi \mu _k}{z}\right) \nonumber \\&\quad +\sum _{k=1}^L \mathop {{\mathrm{Res}}}\left( \frac{\Gamma (n+w)}{\Gamma (w)}\,\chi (w)\,z^{-n-w},w=s_k \right) \end{aligned}$$

#### *Proof*

(i) Indeed, we have
  34 $$\begin{aligned} \begin{aligned} 2\sum _{k=1}^{\infty } \frac{\alpha _k}{\lambda _k^s}\,e^{-{(z\lambda _k)}^2}&=2\,\,{\left( z^{-1}\right) }^{r-s}\sum _{k=1}^{\infty } \beta _k \ {}_1\Psi _1\left( \begin{array}{c} (\frac{r-s}{2},1) \\ (\frac{r}{2},1) \end{array}\,; -{\left( \frac{\mu _k}{z}\right) }^2\right) \\&\quad +\sum _{k=1}^L {\mathop {{\mathrm{Res}}}}\left( \frac{\Gamma \left( -\frac{s}{2}+\frac{w}{2}\right) }{\Gamma \left( \frac{w}{2}\right) } \,\chi (w)\,z^{s-w},w=s_k \right) \end{aligned}\end{aligned}$$
(ii) This is a special case of (29) with $B=C=1$, $b=0$ and then one needs to appeal to (3).
(iii) This is the special case of Theorem 5 with $B=C=1$, $b=0$. We choose $s=-n$ and appeal to the well-known relation
  35 $$\begin{aligned}&L_{n}^{\alpha }(z)=\frac{{(-1)}^n}{n!}\Psi (-n,\alpha +1;z) =\frac{{(\alpha +1)}_n}{n!}\Phi (-n,\alpha +1;z) \nonumber \\&\quad ={}_1F_1\left( \begin{array}{c} -n \\ \alpha +1 \end{array};z \right) . \end{aligned}$$
  The proof will be completed now on noting the relation between the Pochhammer symbol ${\alpha }_n=\alpha (\alpha +1)\cdots (\alpha +n-1)$ and the gamma function
  $$\begin{aligned} \Gamma (-n-\alpha )=\frac{{(-1)}^n}{{(\alpha +1)}_n} \end{aligned}$$
  for integer $$n\ge 0$$.

$$\square$$

The special case of (32) with $s=0$ is a formula due to Bochner (1958) [which was first proved in Bochner and Chandrasekharan (1956)] and was revisited by Berndt (1970).

#### *Remark 8*

It may be appropriate to make some comments on the characterization of the Riemann zeta- and allied functions from the functional equation of the Riemann type in reminiscence of H. Hamburger who made the first characterization of the Riemann zeta-function. The authors of Bochner and Chandrasekharan (1956), Chandrasekharan and Mandelbrojt (1959) and Chandrasekharan and Mandelbrojt (1957) are concerned with bounding the number of linearly independent solutions to the functional Eq. (19), thus leading to the uniqueness of the solution. In all these investigations a special case of (31) plays an essential role, which in turn was suggested by Siegel’s simplest proof Siegel (1922) of Hamburger’s theorem. Here one sees the importance of exhausting those relations that are equivalent to the functional equation.

### Atkinson–Berndt Abel mean

#### *Example 9*

(Atkinson–Berndt Abel mean) Berndt (1970) proved the following extension of Atkison’s (1950) result.

Let $$\chi$$ has at most simple poles (for simplicity) at $$s_k$$ with residue $$\rho _k$$ ($$1 \le k \le L$$) respectively. Let us also assume that the weighted Lambert series (for every $$\delta >0$$$$(\mathop {{\mathrm{Re}}} \delta >0)$$)

$$\begin{aligned} \tilde{\varphi }(s, \delta )=\sum _{k=0}^\infty \frac{\alpha _k}{\lambda _k^{s}}\,e^{-\lambda _k \delta } \end{aligned}$$

converges for $\sigma >\sigma _0$, where $\sigma _0 \le \sigma _{\varphi }$. Then for $\sigma >\sigma _0$ and for $s \ne s_k$,

36 $$\begin{aligned} \lim _{\delta \rightarrow 0}\left\{ \tilde{\varphi }(s,\delta )- \sum _{k=1}^L \frac{\rho _k\Gamma (s_k-s)}{\Gamma (s_k)}\delta ^{s-s_k}\right\} =\varphi (s). \end{aligned}$$

Here the limit $\delta \rightarrow 0$ means $\delta \rightarrow +0$ inside $\mathop {{\mathrm{Re}}} \delta >0$, which therefore suggests the name ‘Abel mean’. He deduced (36) by computing

37 $$\begin{aligned} \lim _{\delta \rightarrow 0}\left\{ \Gamma (s)\tilde{\varphi }(s,\delta ) -\int _0^1 x^{s-1}P(x+\delta )\,{\mathrm{d}}x\right\} \end{aligned}$$

in two different ways.

We shall use Theorem 5 to show that (next corollary) the Atkinson–Berndt Abel mean is nothing but another way to prove the functional equation.

#### **Corollary 10**

*The Atkinson–Berndt Abel mean* (36) *for*$\sigma <\min \{r,r-\sigma _{\psi }\}$*leads to the counterpart of the functional equation* (21):

38 $$\begin{aligned} \lim _{\delta \rightarrow 0}\left\{ \tilde{\varphi }(s,\delta )- \sum _{k=1}^L \frac{\rho _k\Gamma (s_k-s)}{\Gamma (s_k)}\delta ^{s-s_k}\right\} =\frac{\Gamma (r-s)}{\Gamma (s)}\psi (r-s) \end{aligned}$$

#### *Proof*

Indeed, for $\sigma >\sigma _{\varphi }$ (and *r*/2), we may take the limit in two different ways. Now using Theorem 5 we get,

39 $$\begin{aligned} \begin{aligned} \tilde{\varphi }(s,\delta )={}&\delta ^{s-r}\frac{\Gamma (r-s)}{\Gamma (r)}\sum _{k=1}^{\infty } b_k {}_1F_{1}\left( \begin{array}{c}r-s \\ r \end{array};-\frac{\mu _k}{\delta }\right) \\&+\sum _{k=1}^L \frac{\Gamma (s_k-s)}{\Gamma (s_k)}\rho _k\delta ^{s-s_k}. \end{aligned}\end{aligned}$$

Now we apply (Erdélyi et al. 1953, (3), p. 276)

$$\begin{aligned} \Phi (a,c;x)=\frac{\Gamma (c)}{\Gamma (c-a)}(-x)^{-a}\left\{ 1+O\left( |x|^{-1}\right) \right\} , \; \mathop {{\mathrm{Re}}}x \rightarrow -\infty \end{aligned}$$

to (39) to deduce (38) for $\sigma <\min \{r,r-\sigma _{\psi }\}$. $\square$

### The $H^{2,1}_{1,2}\leftrightarrow H^{2,1}_{1,2}$ formula

We state the symmetric version of Theorem 2 (complementing it) since it still contains the incomplete gamma expansions.

#### **Theorem 11**

*Zeta-functions that satisfy the functional equation* (19) *have a symmetric expansion*$H_{1,2}^{1,2}\leftrightarrow H_{1,2}^{1,2}$*which is equivalent to* (19) *and is*

40 $$\begin{aligned}&\sum _{k=1}^{\infty } \frac{\alpha _k}{\lambda _k^s}\,H_{1,2}^{2,1}\left( z \lambda _k \left| \begin{array}{c} (1-a,A) \\ \left( Cs,C\right) ,(b,B) \end{array} \right. \right) \nonumber \\&= \sum _{k=1}^{\infty } \frac{\beta _k}{\mu _k^{r-s}}\,H_{1,2}^{2,1}\left( \frac{\mu _k}{z} \left| \begin{array}{c} (1-b,B) \\ \left( C(r-s),C\right) ,(a,A) \end{array} \right. \right) \nonumber \\&\quad +\sum _{k=1}^L {\mathrm{Res}}\Big (\Gamma (b-Bs+Bw)\Gamma (a+As-Aw)\,\chi (w)\,z^{s-w},w=s_k \Big ). \end{aligned}$$

Its special case takes the shape,

41 $$\begin{aligned} \begin{aligned}&\frac{1}{C}\sum _{k=1}^{\infty } \frac{\alpha _k}{\lambda _k^s} \,G_{1,2}^{2,1}\left( {(z\lambda _k)}^{\frac{1}{C}}\left| \begin{array}{c} a \\ Cs,b \end{array} \right. \right) \\&=\frac{1}{C}\sum _{k=1}^{\infty } \frac{\beta _k}{\mu _k^{r-s}} \,G_{1,2}^{2,1}\left( {\left( \frac{\mu _k}{z}\right) }^{\frac{1}{C}}\left| \begin{array}{c} 1-b \\ C(r-s),1-a \end{array} \right. \right) \\&\quad +\sum _{k=1}^L {\mathop {{\mathrm{Res}}}}\left( \Gamma (b-C(s-w))\Gamma (a+C(s-w))\,\chi (w)\,z^{s-w},w=s_k\right) . \end{aligned} \end{aligned}$$

An application of (41) entails confluent hypergeometric expansion.

#### **Proposition 12**

42 $$\begin{aligned}&\frac{1}{C}\,\Gamma (a+b)\,\Gamma \left( a+Cs\right) z^s\sum _{k=1}^{\infty } \alpha _k\, U\Big (a+Cs,1-b+Cs,{(z\lambda _k)}^{\frac{1}{C}}\Big ) \nonumber \\&\quad =\frac{1}{C}\,\Gamma (a+b)\,\Gamma \left( b+C(r-s)\right) {\left( \frac{1}{z}\right) }^{r-s} \nonumber \\&\sum _{k=1}^{\infty } \beta _k\, U\left( b+C(r-s),1-a+C(r-s),{\left( \frac{\lambda _k}{z}\right) }^{\frac{1}{C}}\right) \nonumber \\&\quad +\sum _{k=1}^L {\mathop {{\mathrm{Res}}}}\Big (\Gamma \left( b-C(s-w)\right) \Gamma \left( a+C(s-w)\right) \chi (w)\,z^{s-w},w=s_k \Big ). \end{aligned}$$

*This entails the symmetric incomplete gamma series:*

43 $$\begin{aligned} \begin{aligned}&\Gamma \left( 1-b+Cs\right) z^s\sum _{k=1}^{\infty } \alpha _k e^{{(z\lambda _k)}^{1/C}} \,\Gamma \left( b-Cs,{(z\lambda _k)}^{1/C}\right) \\&=\Gamma \left( b+C(r-s)\right) {\left( \frac{1}{z} \right) }^{r-s} \sum _{k=1}^{\infty } \beta _k e^{{\left( \frac{z}{\mu _k}\right) }^{1/C}} \Gamma \left( 1-b-C(r-s),{\left( \frac{\mu _k}{z}\right) }^{1/C}\right) \\&\quad +C\sum _{k=1}^L {\mathop {{\mathrm{Res}}}}\left( \Gamma \left( b-C(s-w)\right) \Gamma \left( 1-b+C(s-w)\right) \chi (w)\,z^{s-w},w=s_k \right) \end{aligned} \end{aligned}$$

#### *Proof*

(42) follows from (41) in view of (13) and (11).

(43) is a specification of (42) with $a=1-b$ in view of (14). $\square$

We will now work out few examples:

#### *Example 13*

In the familiar set up of $\zeta (s)$ the relation (42) can be rewritten as the sum over the full lattice ${\mathbb {Z}}$ as:

44 $$\begin{aligned} \begin{aligned}&\Gamma \left( a+\tfrac{s}{2}\right) \,\sum _{k\in {\mathbb {Z}}} U\Big (a+\tfrac{s}{2},1-b+\tfrac{s}{2},z^2\pi k^2\Big )\\&\quad =\frac{1}{\,z\,}\,\Gamma \left( b+\tfrac{1-s}{2}\right) \,\sum _{k\in {\mathbb {Z}}} U\left( b+\tfrac{1-s}{2},1-a+\tfrac{1-s}{2},\frac{\pi k^2}{z^2}\right) . \end{aligned}\end{aligned}$$

Here the residual function is

45 $$\begin{aligned} {\mathrm{P}}(z)&=z^{s-1}\,\Gamma \left( b+\tfrac{1-s}{2}\right) \Gamma \left( a-\tfrac{1-s}{2}\right) \nonumber \\&\quad -z^s\,\Gamma \left( a+\tfrac{s}{2}\right) \Gamma \left( b-\tfrac{s}{2}\right) . \end{aligned}$$

Thus one re-discovers (Erdélyi et al. 1953, I,(2) p. 255),

$$\begin{aligned} U(a,b;z) =\frac{1}{\Gamma (a)} \int _0^\infty e^{tz} t^{a-1}(1+t)^{b-a-1}\,{\mathrm {d}}t, \quad \mathop {{\mathrm{Re}}} a>0. \end{aligned}$$

Whence,

$$\begin{aligned} U(a,b;0) =\frac{1}{\Gamma (a)} \int _0^\infty t^{a-1}(1+t)^{b-a-1}\,{\mathrm {d}}t. \end{aligned}$$

This is another form of the integral expression for the beta-function ${\mathrm{B}}(a,b-a-1)=\frac{\Gamma (a)}{\Gamma (b-a-1)}$. Thus we have

$$\begin{aligned} U(a,b;0)=\frac{\Gamma (1-b)}{\Gamma (1+a-b)}. \end{aligned}$$

Hence (42) after dividing by $\Gamma (a+b)$ and moving one term in (45) to the left entails:

$$\begin{aligned} \begin{aligned}&2\,\Gamma \left( a+\tfrac{s}{2}\right) \,\sum _{k=1}^\infty U\Big (a+\tfrac{s}{2},1-b+\tfrac{s}{2},z^2\pi k^2\Big )\\&\quad \quad +\Gamma \left( a+\tfrac{s}{2}\right) U\Big (a+\tfrac{s}{2},1-b+\tfrac{s}{2},0\Big )\\&\quad =\frac{2}{\,z\,}\,\Gamma \left( b+\tfrac{1-s}{2}\right) \,\sum _{k=1}^\infty U\left( b+\tfrac{1-s}{2},1-a+\tfrac{1-s}{2},\frac{\pi k^2}{z^2}\right) \\&\quad \quad +\frac{1}{\,z\,}\,\Gamma \left( b+\tfrac{1-s}{2}\right) U\left( b+\tfrac{1-s}{2},1-a+\tfrac{1-s}{2},0\right) . \end{aligned} \end{aligned}$$

#### *Example 14*

As in Example 13 one can easily show that in the case of the Riemann zeta-function, (43) may be written as

$$\begin{aligned}\begin{aligned}&\Gamma \left( 1-b+\tfrac{s}{2}\right) \,\sum _{k\in {\mathbb {Z}}} \Gamma \Big (b-\tfrac{s}{2},z^2\pi k^2\Big )\,e^{z^2\pi k^2}\\&\quad =\frac{1}{\,z\,}\,\Gamma \left( b+\tfrac{1-s}{2}\right) \,\sum _{k\in {\mathbb {Z}}} \Gamma \left( 1-b-\tfrac{1-s}{2},\frac{\pi k^2}{z^2}\right) e^{\frac{\pi k^2}{z^2}}. \end{aligned} \end{aligned}$$

#### *Example 15*

(Ueno–Nishizawa formula Lavrik 1968; Ueno and Nishizawa 1995) Theorem 11 entails the incomplete gamma expansion due to Ueno and Nishizawa (1995).

46 $$\begin{aligned} \begin{aligned}&2\sqrt{\pi }\,\Gamma (s)\,z^s\sum _{k=1}^{\infty } \frac{\alpha _k}{\big (2z\lambda _k+1\big )^s} \\&=\Gamma (s)\sum _{k=1}^{\infty } \frac{\beta _k}{\mu _k^{1-s}} \left( e^{-\frac{1-s}{2}\pi i +\frac{\mu _k}{z}\,i} \, \Gamma \left( 1-s,\frac{\mu _k}{z}\,i\right) \right. \\&+\left. e^{\frac{1-s}{2}\pi i -\frac{\mu _k}{z}\,i} \, \Gamma \left( 1-s,-\frac{\mu _k}{z}\,i \right) \right) \\&\quad +\sum _{k=1}^L {\mathop {{\mathrm{Res}}}}\Big (\Gamma \left( \tfrac{w}{2}+\tfrac{1}{2}\right) \Gamma (s-w)\,\chi (w)\,z^{s-w},w=s_k\Big ). \end{aligned} \end{aligned}$$

In case of $\zeta (s,w)$ the relation (46) amounts to

47 $$\begin{aligned} \begin{aligned} \zeta (s,w)&=\sum _{k=1}^\infty \left( \frac{e^{-2\pi kwi}}{\left( 2\pi k\,e^{-\frac{\pi i}{2}}\right) ^{1-s}}\,\Gamma \Big (1-s,-2\pi kwi\Big )\right. \\&\quad +\left. \frac{e^{2\pi kwi}}{\left( 2\pi k\,e^{\frac{\pi i}{2}}\right) ^{1-s}}\,\Gamma \Big (1-s,2\pi kwi\Big )\right) \\&\quad +\frac{1}{2\,w^{s}}+\frac{1}{w^{s-1}}\,\frac{1}{s-1};\quad 0<w=\frac{1}{2\sqrt{\pi }\,z}<1. \end{aligned} \end{aligned}$$

Now we set $a=0$, $A=1$, $b=\frac{s}{2}+\frac{1}{2}$ and $B=\frac{1}{2}$ in Theorem 11 and use,

48 $$\begin{aligned} \begin{aligned}&H^{2,1}_{1,2}\left( z \left| \begin{array}{@{}c@{}} (1,1)\\ \left( s,\frac{1}{2}\right) ,\left( s+\frac{1}{2},\frac{1}{2}\right) \end{array} \right. \right) =\sqrt{\pi }\,2^{1-2s}\,H^{1,1}_{1,1}\left( 2z \left| \begin{array}{@{}c@{}} (1,1)\\ (2s,1) \end{array} \right. \right) \\&\quad =\sqrt{\pi }\,2^{1-2s}\,G^{1,1}_{1,1}\left( 2z \left| \begin{array}{@{}c@{}} 1\\ 2s \end{array} \right. \right) =2\sqrt{\pi }\,\Gamma (2s)\,\frac{z^{2s}}{\big (1+2z\,\big )^{2s}}. \end{aligned} \end{aligned}$$

Again using (8), (5), (10) alongwith (48) we get,

$$\begin{aligned}&H^{2,1}_{1,2}\left( z \left| \begin{array}{@{}c@{}} \left( s,\frac{1}{2}\right) \\ \left( s,\frac{1}{2}\right) ,(0,1) \end{array} \right. \right) \\&\quad =H^{3,2}_{3,4}\left( z \left| \begin{array}{@{}c@{}} \left( s,\frac{1}{2}\right) ,\left( s+\frac{1}{2},\frac{1}{2}\right) ,\left( s+\frac{1}{2},\frac{1}{2}\right) \\ \left( s,\frac{1}{2}\right) ,\left( s+\frac{1}{2},\frac{1}{2}\right) ,(0,1),\left( s+\frac{1}{2},\frac{1}{2}\right) \end{array} \right. \right) \\&\quad =2\pi \,H^{2,1}_{2,3}\left( z \left| \begin{array}{@{}c@{}} (2s,1),\left( s+\frac{1}{2},\frac{1}{2}\right) \\ (2s,1),(0,1),\left( s+\frac{1}{2},\frac{1}{2}\right) \end{array} \right. \right) \\&\quad =\frac{1}{i}\left\{ e^{(s+\frac{1}{2})\pi i}\,H^{2,1}_{1,2}\left( e^{-\frac{\pi }{2} i}z \left| \begin{array}{@{}c@{}} (2s,1)\\ (2s,1),(0,1) \end{array} \right. \right) \right. \\&\quad \quad -\left. e^{-(s+\frac{1}{2})\pi i}\,H^{2,1}_{1,2}\left( e^{\frac{\pi }{2} i}z \left| \begin{array}{@{}c@{}} (2s,1)\\ (2s,1),(0,1) \end{array} \right. \right) \right\} \\&\quad =\frac{1}{i}\left\{ e^{(s+\frac{1}{2})\pi i}\,G^{2,1}_{1,2}\left( e^{-\frac{\pi }{2}i}z \left| \begin{array}{@{}c@{}} 2s\\ 2s,0 \end{array} \right. \right) -e^{-(s+\frac{1}{2})\pi i}\,G^{2,1}_{1,2}\left( e^{\frac{\pi }{2}i}z \left| \begin{array}{@{}c@{}} 2s\\ 2s,0 \end{array} \right. \right) \right\} \\&\quad =\Gamma (1-2s) \Big (e^{-s\pi i+zi}\,\Gamma \big (2s,zi\big )+e^{s\pi i-zi}\,\Gamma \big (2s,-zi\big )\Big ). \end{aligned}$$

#### *Remark 16*

(Hurwitz’s formula) We note that using the Fourier expansion for the Dirac delta function as in (Kanemitsu and Tsukada 2007, p. 75), the Ueno–Nishizawa formula (47) (Ueno and Nishizawa 1995) leads us to the familiar Hurwitz formula

$$\begin{aligned} \zeta (s,w)=\frac{\Gamma (1-s)}{(2\pi )^{1-s}} \left( e^{\frac{1-s}{2}\,\pi i}\,l_{1-s}(-w)+e^{-\frac{1-s}{2}\,\pi i}\,l_{1-s}(w)\right) , \end{aligned}$$

where $l_s(w)$ is the Lerch zeta-function.

## Conclusions

We have shown that the modular relation which is an equivalent expression of the functional equation of the zeta-function in terms of the *H*- and *G*-functions entails almost all existing Ewald expansion as a hypergeometric function hierarchy. Especially, we deduce all incomplete gamma series from the general modular relation.

## References

[CR1] Atkinson FV (1950). The Riemann zeta-function. Duke Math J.

[CR2] Berndt BC (1969). Identities involving the coefficients of a class of Dirichlet series III. Trans Am Math Soc.

[CR3] Berndt BC (1970). Identities involving the coefficients of a class of Dirichlet series IV. Trans Am Math Soc.

[CR4] Bochner S, Chandrasekharan K (1956) On Riemann’s functional equation. Ann Math (2) 63:336–360. Collected papers of Salomon Bochner, Part II, Am Math Soc, Providence, RI 1991, 715–739

[CR5] Bochner S (1958). On Riemann’s functional equation with multiple gamma factors. Ann Math (2).

[CR6] Chandrasekharan K, Mandelbrojt S (1959). On Riemann’s functional equation. Bull Am Math Soc.

[CR7] Chandrasekharan K, Mandelbrojt S (1957). On Riemann’s functional equation. Ann Math (2).

[CR8] Erdélyi A, Magnus W, Oberhettinger F, Tricomi FG (1953) Higher transcendental functions, vols I–III, Based, in part, on notes left by Harry Bateman. McGraw-Hill, New York

[CR9] Kanemitsu S, Tanigawa Y, Yoshimoto M (2002) Ramanujan’s formula and modular forms, number-theoretic methods—future trends. In: Kanemitsu S, Jia, C (eds) Proceedings of a conference held in Iizuka. Kluwer, Dordrecht, pp 159–212

[CR10] Kanemitsu S, Tsukada H (2007). Vistas of special functions.

[CR11] Kanemitsu S, Tsukada H (2014). Contributions to the theory of zeta-functions: the modular relation supremacy.

[CR12] Lavrik AF (1968) An approximate functional equation for the Dirichlet $$L$$-function. Trudy Moskov Mat Obšč 18:91–104. Trans Moscow Math Soc, 18 (1968), 101–115

[CR13] Prudnikov AP, Bychkov YuA, Marichev OI (1986). Integrals and series, supplementary chapters.

[CR14] Siegel CL (1922) Bemerkungen zu einem Satz von Hamburger über die Funktionalgleichung der Riemannschen Zetafunktion. Math Ann 85:276–279. Ges Abh, Bd I, Springer Verl

[CR15] Szegö G (1926). Beiträge zur Theorie der Laguerreschen Polynome. Zahlentheoretische Anwendungen. Math Z.

[CR16] Tsukada H (2007) A general modular relation in analytic number theory. Number theory: sailing on the sea of number theory. In: Proceedings of the 4th China-Japan seminar on number theory 2006 . World Scitific, Singapore, pp 214–236

[CR17] Ueno K, Nishizawa M (1995) Quantum groups and zeta-functions, in quantum groups: formalism and applications. In: Lubkierski et al J (eds) Proceedings of the Thirtieth Karpacz Winter School (Karpacz, 1994). Polish Sci. Publ. PWN, Warsaw, pp 115–126

